# A Clinical Lecture1Delivered February 3, 1908.

**Published:** 1908-06

**Authors:** Ralph Waldo

**Affiliations:** Professor of Diseases of Women, New York Post-Graduate Medical School and Hospital; Gynecologist to Lebanon Hospital


### A Clinical Lecture[1]

### Case I.—Severe Menorrhagia and Metorrhagia. Case II.—Sterility Probably Due to Long Conical Cervix and Anteflexion.

By RALPH WALDO, M D.

Professor of Diseases of Women, New York Post-Graduate Medical School and Hospital; Gynecologist to Lebanon Hospital.

Case I.—*History.*—The patient is a young woman, 24 years of age, married, Russian. She began to menstruate at the age of fifteen, the periods coming ordinarily every four weeks. The flow continues for five days, a large amount of blood being lost each time. There is some pain at the beginning of the menstrual flow. Five years ago, at the age of nineteen, she married. One year later she gave birth to her only child; delivery normal. No abortions. The patient has not been well since the birth of this child. She remained in the hospital three weeks after the birth of child. Six months later she was operated upon in another hospital, where she thinks her right ovary was removed. One year after the first operation she was operated upon again in the same hospital, but does not know the nature of the operation. For the past three months she has been flowing almost continuously, during the first part of which period the flow was very profuse, for the past two or three weeks somewhat less. She experiences no pain in any part of the body. She is pale and anemic, feels weak, has fair appetite; bowels are regular.

*Examination.*—A very simple method of determining whether the abdominal muscles are separated or not is to have the patient, while on the table, to lift her head up. This is much more satisfactory than telling her to bear down; it is also a good way to exercise and develop the abdominal muscles. By this means we find that there is no separation of the muscles in this case.

The right kidney is decidedly movable. The kidney is normally slightly movable, and ordinarily the right kidney lies lower in the abdomen than the left. In a patient thin and anemic, as is this woman, you will nearly always find the kidneys movable, especially the right. Unless there is some special indication,

---

1. Delivered February 3, 1908.

leave the kidney alone. I have never seen a patient benefited by attaching a kidney that is movable within a short range—I am not speaking of floating kidney. If such a patient is built up and her normal adipose restored, the kidney will regain its position.

I cannot lay sufficient stress upon the importance of examining the heart and lungs in women where we are called upon to investigate the uterus. Many times the whole prognosis will be influenced by what is found in these organs. Not infrequently, the pelvic organs cannot all be felt by vaginal examination. There may be an apparently trivial disease of the uterus, perhaps a slight laceration, perhaps a little growth, and the patient may have a serious valvular lesion of the heart, perhaps associated with marked dilatation.

The uterus is soft, has been attached high up, and there is laceration of the cervix. There is no tumor. The patient is suffering from severe menorrhagia and metrorrhagia lasting about three months. There is no condition outside the uterus which would cause it, so we must look for something inside the uterus. It may be due to fungus endometritis; it may be due to the laceration of the cervix, or it may be due to a type of endometritis associated with a small fibroid; on the other hand, it may be the early stage of some very serious lesion, such as carcinoma.

*Treatment.*—The patient should be put under an anesthetic and the inside of the uterus thoroughly explored and curetted, and the scrapings examined microscopically by a competent pathologist. If carcinoma is present in a woman who has bled as long as this patient has, you will find in the uterus a place that is hard, where more or less of a cavity will be left after curettage. In such a case I would advise a sharp curet. If you scrape off the entire endometrium, going down to the muscularis, there is no use putting into the uterus a little strip of gauze to keep open the uterine cavity—it will not do it. There is only one indication for packing the uterus—hemorrhage. If you pack the uterus after an ordinary curettage for endometritis, the patient will have more temperature and more pain than she will if you do not. I cannot see any reason for plugging up the lower end of the cervix after curettage. Nature always leaves the cervix upon where there is anything to be expelled. When I used to pack the uterus following curettage, I noticed that my patients always had a little elevation of temperature, so I decided to try fifty cases with and fifty without packing. I found that those without ran a smoother course than those with it. I am not speaking, of course, of cases with severe hemorrhage. Where there are fungosities, curet and leave alone. There will be more or less drainage, but the uterus is trying all the time to get that out. Wipe out the uterus with gauze. If you have to use an antiseptic douche, always follow it with normal saline or sterile water.

When the patient is in the Sims' position, it does not necessarily mean that the Sims' speculum must be used—you can use any speculum you wish. In a general way the patient is much more easily tamponed in either the Sims' or the knee-chest position. The clothing must always be removed from the abdomen, everything must be loose, and then, with the patient in either of these positions, the pelvic organs gravitate out of the pelvis and the vagina is "ballooned open." The tampon can be well introduced. The urethra and other sensitive parts should be protected by a little pad of cotton. The whole upper portion of the vagina and the vaginal portion of the cervix should be painted with Churchill's iodin, after which a tampon with glycerin containing a mild antiseptic should be introduced.

What antiseptic should be employed? Something to prevent decomposition of the glycerin. An old method, and one as good as anything devised, is to use chemically pure glycerin (Price's glycerin). It is difficult to obtain and expensive. The ordinary glycerin of the market contains a good many foreign substances, particularly fatty acids, and if you do not use some mild antiseptic substance, decomposition will take place and the patient will have vaginitis. Various antiseptics have been used—carbolic acid, boroglycerid, boric acid, borolyptol, and lastly ichthyol which is no better than borolyptol or any of the others. Boric acid, one ounce to the pint of water, is as good as anything and does not soil the patient's limbs.

The tampon must be placed well above the muscular body of the perineum. Allow it to remain in place twenty-four hours, then have the patient remove it and take a hot saline douche. What is a hot douche? It must be 110° to 120° F. If it is not that much, it is not a hot douche. There are patients who will tolerate 120°; most of them will tolerate 115° to 116° 118° after they have used the douche for a little while. By the exercise of a little care you can give the douche much hotter than at first. The external parts should be lubricated with vaselin or cold cream, and the douche-nozzle should be of hard rubber rather than of metal, which conducts the heat so rapidly that it will burn when the water is at a lower temperature.

A practical way of making normal salt solution is to add a heaping teaspoonful of table salt to every two quarts of water. This gives practically a normal salt solution, not accurate enough to inject into the cellular tissue, but sufficiently so for the vaginal douche.

The patient should take the douche properly or not at all. It should be taken at night, on the back, with all the clothes loose. If it is taken twice a day the patient must be instructed to lie down for half an hour or an hour afterward.

In this case the laceration must be repaired if we wish to effect a cure; at the same time the uterus should be curetted.

CASE II.—History. The patient is 22 years of age, married, born in Russia. She began to menstruate at the age of thirteen; menstruates every four weeks; moderate amount of flow, four days duration. Married two years ago, no children, no miscarriages. She has been sick about a year. She complains of pain in the abdomen, chiefly on the left side, pain in the back, and lately she menstruates every three weeks, the flow being scanty and attended with pain; pain precedes the flow. The bowels are constipated; patient anemic; general condition fair. (When I say "somewhat anemic" I mean what my assistant observes. I always examine my patients in private as to the hemoglobin).

*Examination.*—For the benefit of our friends who are very much opposed to the wearing of corsets, I want to call your attention to the bad effect of a lot of tight bands around the waist, as seen in this patient, who does not wear corsets. It is not so marked in her case as in some. I have seen the pelvic organs pushed far down by a lot of tight bands around the waist. I do not say that I advocate corsets, but properly-fitting corsets are of benefit to a woman; improperly-fitting corsets do a great deal of harm. I say this because we hear so much nonsense on the subject. Corsets that fit loosely and properly take the strain off the organs are beneficial; those that constrict the waist and the lower part of the ribs, pushing the pelvic organs down, are harmful.

This patient has never been pregnant, and is very desirous of becoming so. She has anteflexion and a long, conical cervix. Before saying very much about this case, I want to call attention to the fact that in all such cases the semen should be examined for spermatozoa. It is not necessary to go into fine details about the size, etc., of those that will impregnate an ovum—if you find numerous active spermatozoa that is sufficient. That hardly holds, however, in this case, as to whether we should or should not operate. A small uterus, anteflexed, with long, conical cervix, and dysmenorrhea, calls for operative treatment. Ordinary dilatation will not cure the patient. The uterus should be dilated and then Dudley's operation performed. Dudley's operation consists in thoroughly dilating the cervical canal and making an extensive incision through the entire thickness of the posterior lip of the cervix, in the median line, and so turning and suturing the angles left by the incision, that they will not unite to each other. In performing this operation, it is important to extend the incision well up the posterior median line. In this way a direct canal is left.

This method of treatment will cure sterility in a great many cases. I have had marked success with it.

Another kind of sterility, which is sometimes very difficult to cure, is found in women who have given birth to one child. The child is dead, there is another marriage, or for some reason another child is desired. Of course this sterility may be due to the male, but I have never seen statistics giving over 10 per cent. of sterility due to the male, and of this I think it is not over half. If you find a history of double orchitis due to any cause, you will usually find an absence of spermatozoa in the semen of that individual. The spermatozoa may return later, however. The larger percentage of sterility in these cases is due to the woman, and in most cases the seat of the disease is the body, the cervix, the uterus or the tubes. If the patient has anteflexion or stenosis, there will be sterility. Impregnation, however, can take place through a very small opening. The sterility is apt to be due to disease of the cervical endometrium which does not allow the spermatozoon to enter the canal and remain virile. Where there is endometritis there may be a secretion which will destroy the spermatozoon, or the ovum will be very early thrown off after impregnation. Another cause of sterility may be found in the tubes—pyosalpinx or a catarrhal condition. In any case, there is a secretion which destroys the ovum. There may be other causes, such as loss of or disease of the ovaries.

